# Fast Breathing, Faster Thinking: How the Bedside Lung Ultrasound in Emergency (BLUE) Protocol Prevented a Missed Pulmonary Embolism

**DOI:** 10.7759/cureus.108755

**Published:** 2026-05-12

**Authors:** Waleed Khalid Khalafallah Khalid, Hind Abdelazim Mirghani Ibrahim, Ibtisam Ahmed Abdullah Al Hoqani

**Affiliations:** 1 Emergency Medicine, Royal College of Emergency Medicine, London, GBR; 2 Emergency Medicine, Khoula Hospital, Muscat, OMN; 3 Medicine, Ahfad University for Women, Omdurman, SDN

**Keywords:** atypical presentation, bedside ultrasound, blue protocol, deep vein thrombosis, emergency medicine, point-of-care ultrasound, pulmonary embolism, silent pe, tachypnea, vascular ultrasound

## Abstract

Pulmonary embolism is a potentially life-threatening condition with highly variable and often misleading presentations. We report a case of a 51-year-old woman who presented with atypical symptoms, including severe back pain and mild chest discomfort, along with unexplained tachypnea on reassessment, and was ultimately diagnosed with bilateral pulmonary embolism using the bedside lung ultrasound in emergency (BLUE) protocol. Despite the absence of classic features of pulmonary embolism or deep vein thrombosis and preserved blood pressure on presentation, her marked tachypnea prompted a point-of-care ultrasound, which revealed a non-compressible femoral vein. Computed tomography pulmonary angiography confirmed bilateral pulmonary embolism. Laboratory results, which became available only after imaging, further supported the diagnosis. This case highlights the value of integrating point-of-care ultrasound into emergency care, especially in patients with atypical presentations. Ultrasound is truly the modern stethoscope of the emergency physician.

## Introduction

Pulmonary embolism (PE) remains a challenging diagnosis due to its protean clinical presentation. While classic symptoms such as pleuritic chest pain, hemoptysis, or unilateral limb swelling are well described in textbooks and exam scenarios, real-life cases often lack these hallmark features, particularly in patients with underlying comorbidities [1,2]. PE may have a subtle clinical presentation, particularly in patients with overlapping cardiopulmonary conditions such as heart failure or chronic obstructive pulmonary disease (COPD), further complicating diagnosis [3].

Emergency departments (EDs) frequently manage patients with undifferentiated dyspnea or isolated tachypnea. In such scenarios, decision tools such as Wells or Geneva scores and D-dimer testing have variable sensitivity and can delay diagnosis [4]. Clinical gestalt alone may be unreliable, especially when confounded by anchoring bias from recent hospitalizations or presumed alternative diagnoses.

Point-of-care ultrasound (POCUS), particularly the bedside lung ultrasound in emergency (BLUE) protocol, provides a rapid, bedside, non-invasive tool to narrow differential diagnoses in dyspneic patients. It allows for an integrated evaluation of the lungs and venous system, identifying conditions like pneumonia, pneumothorax, pulmonary edema, and PE with high accuracy [5]. Emergency physicians trained in ultrasound can use it as a powerful extension of clinical examination, providing additional diagnostic capabilities for the rapid assessment of acute conditions, and it has increasingly been considered the modern stethoscope of the emergency physician [6].

The BLUE protocol, first described by Lichtenstein and Mezière in 2008, is a structured lung ultrasound approach developed for the rapid evaluation of acute respiratory failure [2]. It is based on the assessment of lung sliding, A-lines, B-lines, and anterior lung consolidations at defined points on the chest wall (BLUE points). Using characteristic ultrasound profiles (A-, B-, and C-profiles), the protocol enables clinicians to rapidly narrow the differential diagnosis at the bedside and differentiate between common causes of respiratory failure, including pulmonary edema, pneumonia, pneumothorax, chronic obstructive pulmonary disease, asthma, and pulmonary embolism. In cases demonstrating an A-profile, further evaluation for deep vein thrombosis is performed; if present, this supports pulmonary embolism, while negative findings prompt assessment of the posterior and/or lateral alveolar and/or pleural syndrome (PLAPS), which points to evaluate for alternative diagnoses such as pneumonia or COPD/asthma.

## Case presentation

A 51-year-old woman with visual impairment presented to the emergency department (ED) in a wheelchair with severe lower back pain (pain score 10/10) and mild chest discomfort. Her daughter reported a recent hospitalization for a suspected demyelinating disorder, which was investigated with lumbar punctures and treated with high-dose methylprednisolone. The hospital course was complicated by heparin-induced thrombocytopenia (HIT). Her medical history included hypertension and type 2 diabetes mellitus.

On arrival, the patient’s main concern was the severe back pain. The chest discomfort had resolved with analgesia and was not initially emphasized. There was no history of limb swelling, fever, hemoptysis, or neurological deficits. Vital signs showed a heart rate of 110 beats per min, blood pressure of 100/75 mmHg, oxygen saturation of 94%, and a respiratory rate documented as “normal” in triage. However, manual reassessment revealed a respiratory rate of 31 breaths per min. An electrocardiogram showed sinus tachycardia.

**Figure 1:**
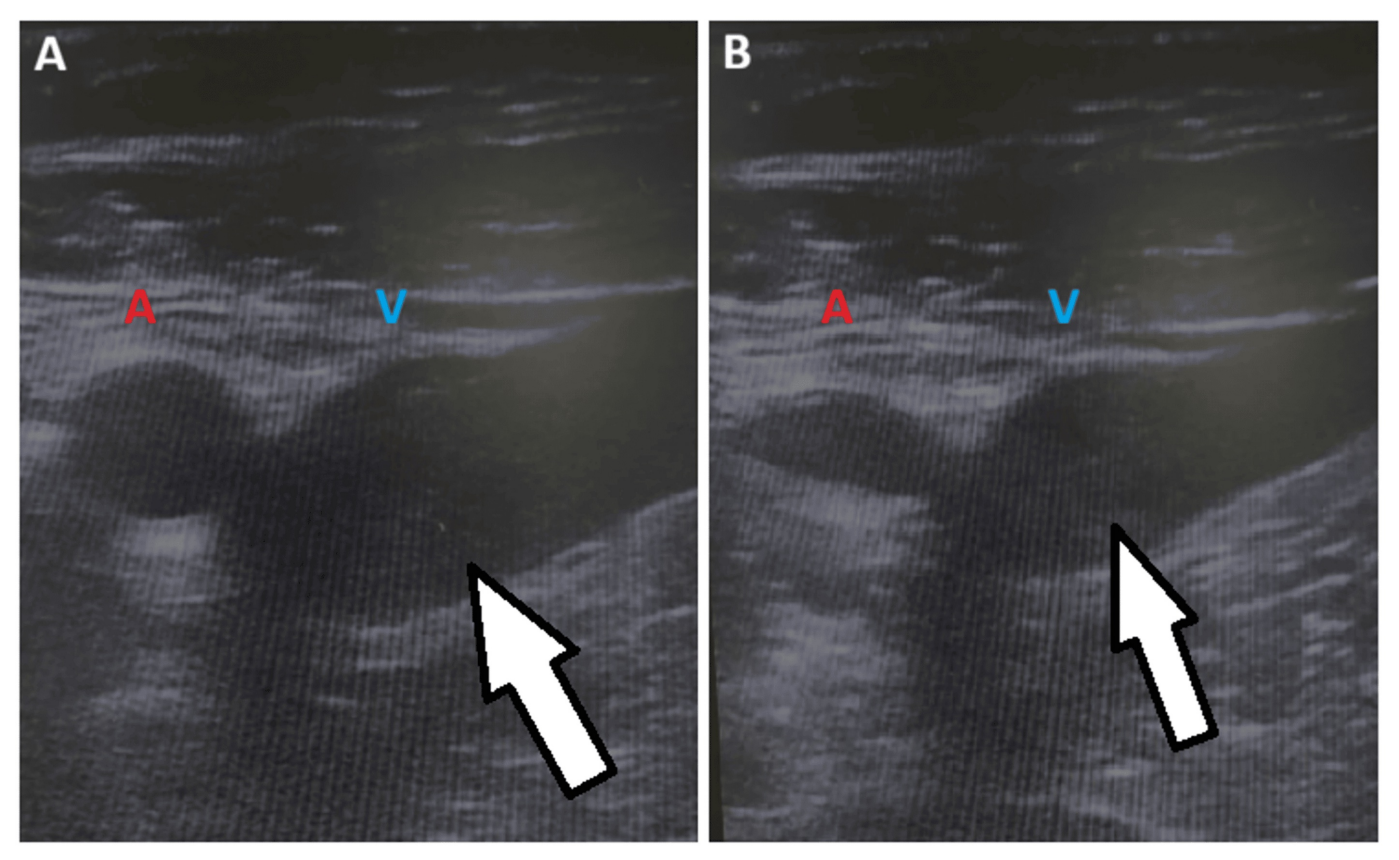
Right lower limb compression ultrasound showing DVT. Compression ultrasound of the right common femoral vein. The arrow indicates an intraluminal echogenic thrombus. A denotes the artery, and V denotes the vein. In panel A (before compression), both vessels are visualized. In panel B (during compression), the artery collapses normally, while the vein remains non-compressible, consistent with deep vein thrombosis. DVT: deep vein thrombosis

**Figure 2:**
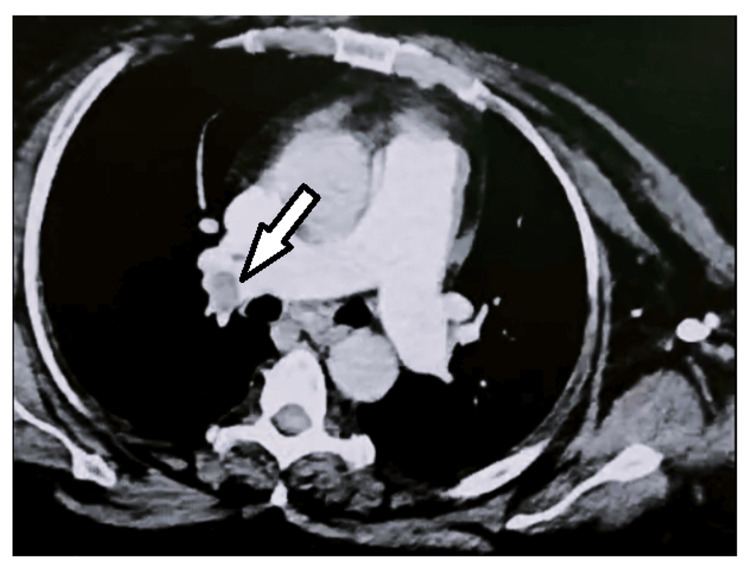
CT pulmonary angiography showing bilateral PE. Axial CTPA image demonstrating filling defects in the right main pulmonary artery and left lower lobe pulmonary artery, consistent with bilateral pulmonary embolism. The arrow indicates an obvious thrombus within the right pulmonary artery. PE: pulmonary embolism; CTPA: computed tomography pulmonary angiography

Despite the family attributing her back pain to the recent lumbar puncture, the elevated respiratory rate without an obvious cause raised clinical suspicion. A full airway, breathing, circulation, disability, exposure (ABCDE) assessment was performed in line with standard emergency care protocols. Chest auscultation revealed mild basal crackles. A focused assessment using the bedside lung ultrasound in emergency (BLUE) protocol was initiated [2]. Lung ultrasound demonstrated an A-profile in all zones. Lower limb compression ultrasound identified a non-compressible right common femoral vein with an intraluminal echogenic thrombus (Figures 1A, 1B). Given these findings, anticoagulation with oral rivaroxaban 15 mg was initiated due to the recent history of heparin-induced thrombocytopenia (HIT), and urgent computed tomography pulmonary angiography (CTPA) confirmed bilateral pulmonary emboli involving the right main and left lower lobe branches (Figure 2).

Subsequent laboratory results, available after bedside ultrasound assessment and CTPA, are summarized in Table 1. These demonstrated elevated inflammatory and thrombotic markers, which, if interpreted in isolation or prior to the BLUE protocol assessment, could have suggested pneumonia or sepsis rather than thromboembolism. Without the preceding bedside ultrasound findings, the laboratory abnormalities may have diverted the clinical impression away from pulmonary embolism.

**Table 1:** Laboratory investigations on initial presentation. Laboratory test results obtained after bedside ultrasound assessment and CTPA. These included elevated inflammatory and thrombotic markers. Reference ranges are provided for the clinical context. CTPA: computed tomography pulmonary angiography

Parameters	Patient value	Reference range
Hemoglobin (Hb)	13.0 g/dL	11.0-14.5 g/dL
White blood cell count (WBC)	16.53×10⁹/L	2.4-9.5×10⁹/L
Platelet count	123×10⁹/L	150-450×10⁹/L
Red blood cell count (RBC)	4.79×10¹²/L	4.1-5.4×10¹²/L
Hematocrit (Hct)	42.4%	34-43%
Mean corpuscular volume (MCV)	88.5 fL	78-95 fL
Mean corpuscular hemoglobin (MCH)	27.1 pg	26-33 pg
Red cell distribution width (RDW)	16.1%	11.5-16.5%
Neutrophils	15.45×10⁹/L	1.0-4.8×10⁹/L
Lymphocytes	0.65×10⁹/L	1.2-3.8×10⁹/L
Eosinophils	0.01×10⁹/L	0-0.5×10⁹/L
Monocytes	0.40×10⁹/L	0.1-1.3×10⁹/L
Basophils	0.02×10⁹/L	0-0.2×10⁹/L
Mean corpuscular Hb concentration (MCHC)	30.7 g/dL	31-35 g/dL
Mean platelet volume (MPV)	12.5 fL	7-10.5 fL
D-dimer	5.98 mg/L	0-0.5 mg/L
Troponin T	31.180 ng/L	0-14 ng/L
C-reactive protein (CRP)	213.34 mg/L	0-5 mg/L
Glucose	9.90 mmol/L	3.9-7.8 mmol/L
Lactate	3.50 mmol/L	0.5-2.2 mmol/L

The patient was admitted and treated with anticoagulation, intravenous antibiotics, and analgesia. During her hospital stay, further workup, including transesophageal echocardiography, led to a diagnosis of mitral valve infective endocarditis, which was managed according to standard protocol. She remained hospitalized for three weeks and was eventually discharged in stable condition.

Two weeks later, she re-presented to the ED with shortness of breath and was initially treated for presumed heart failure with diuretics. No BLUE protocol or bedside ultrasound was performed during that visit. After two days of worsening symptoms, a repeated CTPA revealed recurrent pulmonary embolism, which was then managed appropriately. She was discharged safely thereafter.

## Discussion

**Figure 3:**
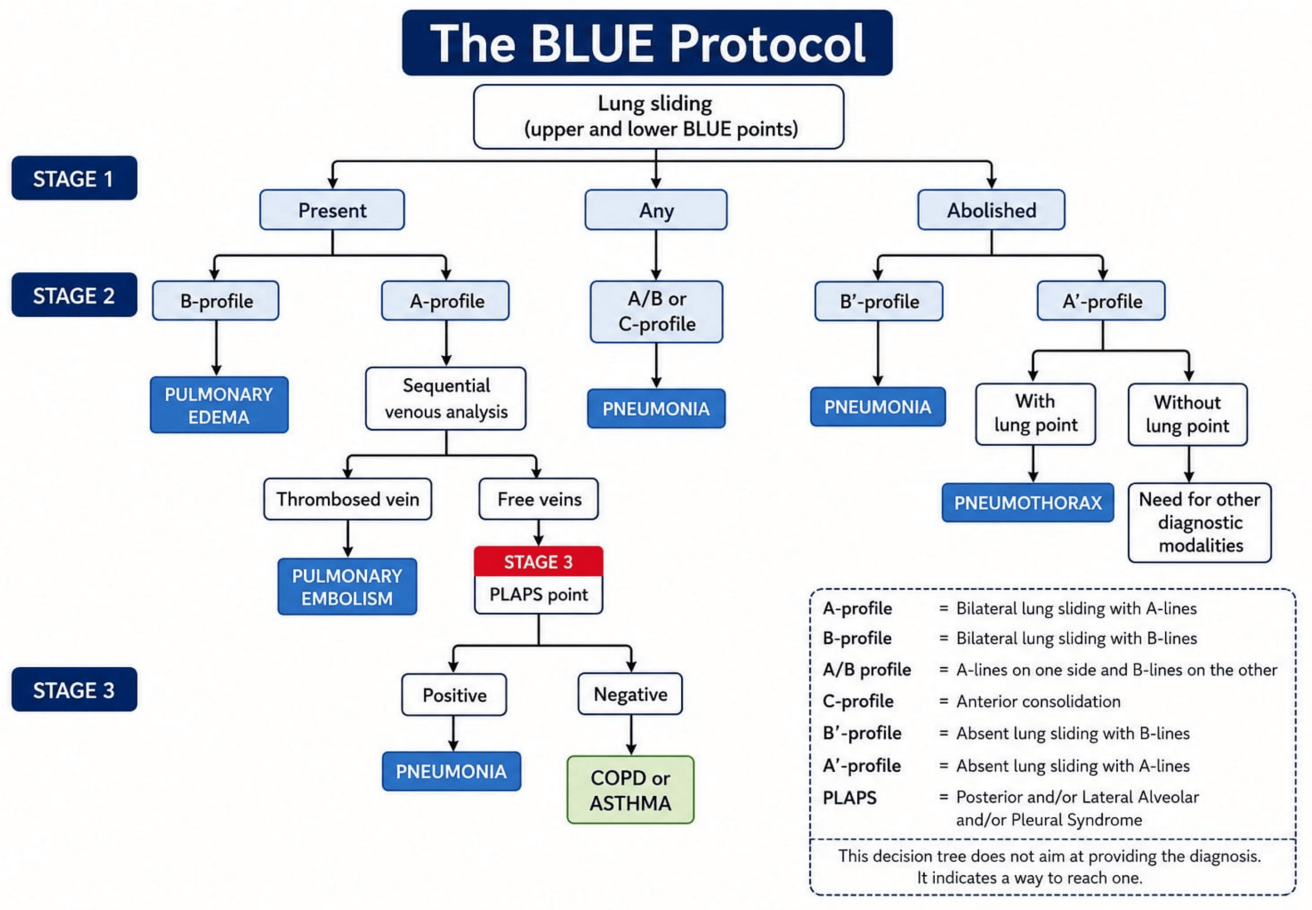
BLUE protocol algorithm for the assessment of acute respiratory failure. Schematic representation of the BLUE protocol demonstrating a stepwise approach to evaluating acute respiratory failure using lung ultrasound profiles (A-, B-, and C-profiles), lung sliding assessment, and targeted venous analysis. The image was created by the authors of this study using Microsoft PowerPoint (Redmond, WA: Microsoft Corp.). BLUE: bedside lung ultrasound in emergency; PLAPS: posterior and/or lateral alveolar and/or pleural syndrome; COPD: chronic obstructive pulmonary disease

This case highlights the diagnostic value of the BLUE protocol in detecting pulmonary embolism in the emergency department, particularly in atypical and undifferentiated presentations (Figure 3). The patient’s back pain, recent hospitalization for neurologic investigation and treatment, and history of heparin-induced thrombocytopenia (HIT) provided multiple potential distractions. Anchoring bias and reliance on triage respiratory rates could have delayed a potentially life-saving diagnosis and treatment.

Manual identification of tachypnea and the decision to perform a BLUE protocol-based lung and venous ultrasound significantly altered this patient’s clinical trajectory. The protocol efficiently ruled out pneumonia, pulmonary edema, and pneumothorax, while pointing toward pulmonary embolism through the detection of deep vein thrombosis. This underscores how ultrasound can expedite diagnosis, even before laboratory results become available.

The later diagnosis of mitral valve infective endocarditis may also have contributed to the thromboembolic risk in this patient. Infective endocarditis is associated with systemic inflammation, endothelial activation, and coagulation pathway dysregulation, resulting in a prothrombotic state that may predispose to venous thromboembolism. Additional contributing factors in this case included recent hospitalization, high-dose corticosteroid therapy, and a history of heparin-induced thrombocytopenia. While pulmonary embolic phenomena are classically associated with right-sided infective endocarditis through septic pulmonary emboli, this patient had mitral valve involvement together with documented lower-limb deep vein thrombosis, making thromboembolic pulmonary embolism the more likely mechanism.

Patient presentations in emergency settings are often complex and non-specific, creating significant diagnostic uncertainty. Emergency physicians must therefore be prepared to assess vague, overlapping, and potentially misleading clinical features, as common presentations may obscure serious or life-threatening underlying conditions. In this context, the BLUE protocol provides a standardized yet adaptable diagnostic framework that can be rapidly deployed in time-sensitive emergency presentations, facilitating earlier recognition and management of potentially life-threatening conditions.

Multiorgan point-of-care ultrasound (POCUS) has demonstrated high diagnostic accuracy in pulmonary embolism. Nazerian et al. reported a sensitivity of 90% and specificity of 86% for pulmonary embolism (PE) using multiorgan ultrasound [1]. Similar studies have shown that integrating lung, cardiac, and venous ultrasound improves diagnostic confidence and reduces the need for unnecessary imaging [2-5]. In the present case, the diagnostic pathway primarily relied on BLUE protocol findings in conjunction with venous ultrasound assessment.

According to the 2019 European Society of Cardiology (ESC) guidelines, early mortality risk stratification in acute pulmonary embolism incorporates hemodynamic status, imaging findings, and cardiac biomarkers. In patients without hemodynamic instability, the presence of either elevated cardiac biomarkers or evidence of right ventricular dysfunction is consistent with the intermediate-low-risk category. In this case, the elevated troponin level supported classification within the intermediate-low-risk category according to ESC criteria [7].

Even when a likely cause is apparent, such as back pain following lumbar puncture or acute decompensated heart failure, clinicians should remain vigilant for concurrent life-threatening conditions. The recurrence of pulmonary embolism in this patient and the absence of the BLUE protocol on her second presentation further illustrate how reliance on clinical history or anchoring bias can lead to missed diagnoses.

Routine application of the BLUE protocol, much like the extended focused assessment with sonography in trauma (eFAST), could significantly reduce diagnostic delays in undifferentiated shortness of breath. Repeated use during recurrent patient presentations, particularly in patients with known heart failure or chronic obstructive pulmonary disease, may uncover overlooked deep vein thromboses or pulmonary embolisms hidden behind overlapping symptoms. Finally, this case reiterates the critical importance of manually counting respiratory rate. Inaccurate triage entries can obscure dangerous presentations and delay timely recognition.

## Conclusions

The BLUE protocol provides emergency physicians with a rapid bedside method to identify life-threatening causes of dyspnea, even when classic symptoms are absent. This case highlights the value of integrating POCUS into routine ED evaluation for unexplained tachypnea. Reliance on triage vitals or anchoring bias can delay diagnosis, while manual reassessment and bedside ultrasound can expedite recognition and management. Earlier identification of pulmonary embolism may facilitate timely treatment and improve overall patient care in complex emergency presentations.

## References

[1] Nazerian P, Vanni S, Volpicelli G (2014). Accuracy of point-of-care multiorgan ultrasonography for the diagnosis of pulmonary embolism. Chest.

[2] Lichtenstein DA, Mezière GA (2008). Relevance of lung ultrasound in the diagnosis of acute respiratory failure: the BLUE protocol. Chest.

[3] Zanobetti M, Scorpiniti M, Gigli C (2017). Point-of-care ultrasonography for evaluation of acute dyspnea in the ED. Chest.

[4] Mathis G, Blank W, Reissig A, Lechleitner P, Reuss J, Schuler A, Beckh S (2005). Thoracic ultrasound for diagnosing pulmonary embolism: a prospective multicenter study of 352 patients. Chest.

[5] Kobal SL, Trento L, Baharami S (2005). Comparison of effectiveness of hand-carried ultrasound to bedside cardiovascular physical examination. Am J Cardiol.

[6] Moore CL, Copel JA (2011). Point-of-care ultrasonography. N Engl J Med.

[7] Konstantinides SV, Meyer G, Becattini C (2020). 2019 ESC Guidelines for the diagnosis and management of acute pulmonary embolism developed in collaboration with the European Respiratory Society (ERS): the task force for the diagnosis and management of acute pulmonary embolism of the European Society of Cardiology (ESC). Eur Heart J.

